# BMP-2 Overexpression Augments Vascular Smooth Muscle Cell Motility by Upregulating Myosin Va via Erk Signaling

**DOI:** 10.1155/2014/294150

**Published:** 2014-03-20

**Authors:** Ming Zhang, Min Yang, Li-ping Liu, Wayne Bond Lau, Hai Gao, Man-kun Xin, Li-Xiao Su, Jian Wang, Shu-Juan Cheng, Qian Fan, Jing-Hua Liu

**Affiliations:** ^1^Department of Cardiology, Beijing An Zhen Hospital, Capital Medical University, and Beijing Institute of Heart, Lung and Blood Vessel Disease, Beijing 100029, China; ^2^Department of Cardiology, Beijing Shijitan Hospital, Capital Medical University, Beijing 100038, China; ^3^Department of Nephrology, First Hospital of Tsinghua University, Beijing 100016, China; ^4^Department of Emergency Medicine, Thomas Jefferson University, Philadelphia, PA 19107, USA

## Abstract

*Background.* The disruption of physiologic vascular smooth muscle cell (VSMC) migration initiates atherosclerosis development. The biochemical mechanisms leading to dysfunctional VSMC motility remain unknown. Recently, cytokine BMP-2 has been implicated in various vascular physiologic and pathologic processes. However, whether BMP-2 has any effect upon VSMC motility, or by what manner, has never been investigated. *Methods.* VSMCs were adenovirally transfected to genetically overexpress BMP-2. VSMC motility was detected by modified Boyden chamber assay, confocal time-lapse video assay, and a colony wounding assay. Gene chip array and RT-PCR were employed to identify genes potentially regulated by BMP-2. Western blot and real-time PCR detected the expression of myosin Va and the phosphorylation of extracellular signal-regulated kinases 1/2 (Erk1/2). Immunofluorescence analysis revealed myosin Va expression locale. Intracellular Ca^2+^ oscillations were recorded. *Results.* VSMC migration was augmented in VSMCs overexpressing BMP-2 in a dose-dependent manner. siRNA-mediated knockdown of myosin Va inhibited VSMC motility. Both myosin Va mRNA and protein expression significantly increased after BMP-2 administration and were inhibited by Erk1/2 inhibitor U0126. BMP-2 induced Ca^2+^ oscillations, generated largely by a “cytosolic oscillator”. *Conclusion.* BMP-2 significantly increased VSMCs migration and myosin Va expression, via the Erk signaling pathway and intracellular Ca^2+^ oscillations. We provide additional insight into the pathophysiology of atherosclerosis, and inhibition of BMP-2-induced myosin Va expression may represent a potential therapeutic strategy.

## 1. Introduction

Recent studies demonstrate that BMP-2, a cytokine of the transforming growth factor-*β* superfamily, plays an important role in both physiological and pathophysiological vascular development [1, 2]. Genetically manipulated BMP-2 deficient mice die between days 7 and 10 of life from cardiac defects prior to bone formation, suggesting the significant cardiovascular importance of BMP-2 [3]. Vascular smooth muscle cells (VSMCs) are a significant source of BMP-2 [4]. VSMC migration from the vascular media to the intima is pivotal in atherosclerosis, playing a central role in the genesis of atherosclerotic plaques and restenotic lesions [5, 6].

VSMC migration is dependent upon cellular motility, driven by cycles of actin polymerization, cellular adhesion, and actin-myosin contraction. Myosins are a large family of structurally diverse actin-dependent molecular motors. All myosins utilize energy from ATP hydrolysis to generate force for unidirectional movement along actin filaments and are regarded as the most essential proteins driving cellular migration [7–9]. The myosin superfamily consists of both conventional and unconventional myosins [10, 11]. Found in various organelles, unconventional myosins are involved in RNA and protein transport, cellular movement, signal transduction, cellular morphology maintenance, and membrane trafficking [12].

The unconventional myosin Va is an actin-based motor protein that transports intracellular cargos and can bundle actin* in vitro*. The relationship and function of myosin Va pertaining to cytoskeletal aspects, cellular morphology, filopodia motility, and neurite extension have been reported [13–15]. Recently, myosin Va was implicated in human cancer dissemination [16, 17]. However, the function of myosin Va within cardiovascular disease remains unclear. Whether BMP-2 affects VSMCs migration via myosin Va, and if so, by what mechanism, has never been determined. We investigate the role of BMP-2 as a potential regulator of myosin Vain VSMCs and dissect the involved underlying mechanisms.

## 2. Material and Methods

The study was carried out in accordance with the institutional review board (IRB) approval. The study protocol was approved by the institutional ethics committee and IRB of the Beijing Anzhen Hospital, Affiliate of Capital Medical University.

### 2.1. Cell Culture

Rat vascular smooth muscle cells were primary-cultured via explant method and grown in RPMI-1640 supplemented with 10% FBS, 100 *μ*g/mL penicillin, 100 *μ*g/mL streptomycin, and 2 mM L-glutamine at 37°C in 5% CO_2_ atmosphere. rVSMCs were utilized for experimentation at passage 4–8 [18].

### 2.2. Recombinant Ads

ViralpAV.EX1d-CMV constructs containing the rat BMP-2/myc/IRES/EGFP expression were from Cyagen Biosciences Inc. (Guangzhou, China). HEK293 cells were transfected by viruses via lipofectamine. The culture medium supernatant was collected and purified by double cesium chloride gradient ultracentrifugation [19]. The viruses were titrated by plaque assay in HEK293 cells. Physical viral particle concentration (vp/mL) was determined spectrophotometrically by wavelength (260 nm) absorbance [20]. Viruses were stored at −80°C until use.

### 2.3. Cell Motility

Cells were plated on 60 mm glass microwell dishes and cultured overnight in RPMI-1640 containing 10% FBS. Cellular movements were monitored by Leica SP5 inverted microscopy. Video images were collected by CCD camera (model 3000; Leica) at 15-minute intervals for 6 hours, digitized, and stored as image stacks via Image J 1.41 software (National Institutes of Health, http://rsb.info.nih.gov/ij/). Image stacks were converted to QuickTime movies. Nuclei positions were tracked to quantify cell motility, and velocities were calculated in *μ*mat 15-minute intervals by the same software [21].

### 2.4. Boyden Chamber Assay

A 2 × 10^4^ aliquot of each cell type was plated onto a 24-well BioCoat Invasion Chamber (BD Biosciences, USA) and cultured for 24 hours. Cells were fixed by methanol and stained by crystal violet. Five cell fields were counted at approximately 40-fold magnification [22].

### 2.5. Wounding Assay

Cells were seeded in 35 mm culture dishes (density 2 × 10^5^ cells per well). An incision was made after 24 hours in the central region of confluence in the culture dish. After an additional 48 hours, the dish was carefully washed to remove detached cells. Fresh medium was added. Cultures were observed at the time of incision and after 48 hours. Phase-contrast microscopy pictures were taken of 6 separate fields of the incised region. The distance between two broad edges of cells was measured and analyzed by Leica LAF software.

### 2.6. RNA Extraction, cRNA Preparation, and Gene Chip Array

High quality rat RNA from BMP-2 infected VMSCs and control cells were obtained by gel electrophoresis (18S and 28S bands) and absorbance spectroscopy (240–320 nm). Briefly, 8 *μ*g of total RNA was reverse-transcribed by oligo (dT) primer coupled to a T7 RNA polymerase binding site. Biotinylated complementary RNA (cRNA) was then synthesized from the resulting complementary DNA (cDNA) via T7 polymerase. 25 *μ*g of biotinylated cRNA was randomly sheared and hybridized for 16 hours to Affymetrix gene chips.

The Affymetrix microarrays (Arabidopsis ATH1 genome array) contain 22,810 probe sets, representing approximately 80% of the gene sequences on a single array. Labeling and hybridization on the ATH1 microarrays (one sample per chip) were performed according to manufacturer's instructions (http://www.affymetrix.com/estore/). The probe arrays were scanned and further analyzed with Genespring software (ver 5.0; Silicon Genetics). Normalization per gene and per chip of the log2 values was performed to allow comparison of three independent replicates performed for each experiment set. Genes were considered to be up- or downregulated if the ratio between BMP-2 and control cells was, respectively, greater than 2 or less than 0.5.

### 2.7. RT-PCR

Gene expression was measured by reverse transcription kit (Promega, WI, USA). Briefly, after the RT of 3 *μ*g of total RNA, cDNA was synthesized. The RT products were subjected to PCR with 2720 thermal cycles (abi) and qRT-PCR via real-time PCR system Fast 7500 (abi.) with the primer sets listed in Table 1(a). The cDNA of glyceraldehyde-3-phosphate dehydrogenase (GAPDH) served as internal control. The semiquantitative RT-PCR consisted of 30 cycles of 94°C, 57.5°C, and 72°C (each for 30 seconds).

### 2.8. Western Blot Analysis

Cellular pellets were lysed by RIPA buffer. 30 *μ*g of total protein samples was separated by 10% SDS-PAGE and electrophoretically transferred to PVDF membranes. Membranes were blocked by 5% nonfat milk in TPBS and incubated for 1 hour at room temperature with primary antibody (see Table 1(b)). Secondary antibody HRP-IgG was applied for 1 hour. After three additional TPBS washes, signals were detected by enhanced chemiluminescence (Amersham Bioscience).

### 2.9. Knockdown of Myosin Va, Expression by siRNA

Three specific sequences of small interfering RNA (siRNA) targeting different regions of rat MYO5a mRNA sequence were designed (RiboBio Co., Ltd, China. 124171130126): siRNA-1: 5′-GGAGAAAGACCACAGATTA-3′; siRNA-2: 5′-GAACCTGATTCTAGAACTA-3′; siRNA-3: 5′-GAAGCAATATAGTGGAGAA-3′. VSMCs were transfected after 48 hours of culture with BMP-2 factor (250 *μ*g/mL). Lipofectamine in transfection reagent (8 *μ*L) was added to 100 *μ*L OptiMEM serum-free medium containing 2 nmol/L of each siRNA oligo, incubated for 10 minutes, and added to the 6 cm plate containing 2 mL medium. After 72 hours, the efficacy of myosin Va silencing (i.e., reduction of gene and protein expression) was determined.

### 2.10. Immunofluorescence Analysis

Cells were seeded upon cover slips at the bottom of culture dishes until subconfluence and were then fixed with 5% acetic acid/95% ethanol (v/v) for 20 minutes. To block nonspecific reactions, 5% nonfat milk was added for 30 minutes, and then anti-MYO5a polyclonal antibody (1 : 250 dilutions in PBS) was administered at room temperature for 1 hour. After washing, anti-goat IgG, FITC fluorescein (1 : 800, Jackson) was added at room temperature for 1 hour. DAPI (1 : 2000 in PBS; DABCO, Sigma) stained for total nuclei. Imaging was performed by Leica SP5 laser scanning confocal microscope.

### 2.11. [Ca^2+^]^i^ Determination Assay

Cells were treated with BMP-2 for 48 hours in dye-free media supplemented with 10% FBS, centrifuged prior to resuspension in calcium-free modified Tyrode buffer (145 mM NaCl, 5.6 mM KCl, 100 *μ*M EGTA, 1.0 mM MgCl_2_, 10 mM glucose, and 5.0 mM HEPES, pH 7.2), and incubated for an additional 15 minutes. Fluorescence was measured at room temperature via Leica SP5 confocal imaging system. Two-dimensional confocal images were taken at 1.5 second time intervals. Fluo-4 AM (Dojindo, Japan) was excited at wavelength 488 nm, and emission was detected at 515 nm. Changes in [Ca^2+^]^i^ were expressed as *R* = *F*/*F*_0_, where *R* is resting fluorescence (*F*) divided by normalized fluorescence (*F*_0_).

### 2.12. Statistical Analysis

All data are presented as mean ± SD. The student's two tailed *t*-test compared the difference between two groups. *P* values less than 0.05 were regarded as statistically significant.

## 3. Results

### 3.1. Successful BMP-2 Overexpression by Adenoviral Transfection Increases VSMC Motility

**Figure 1:**
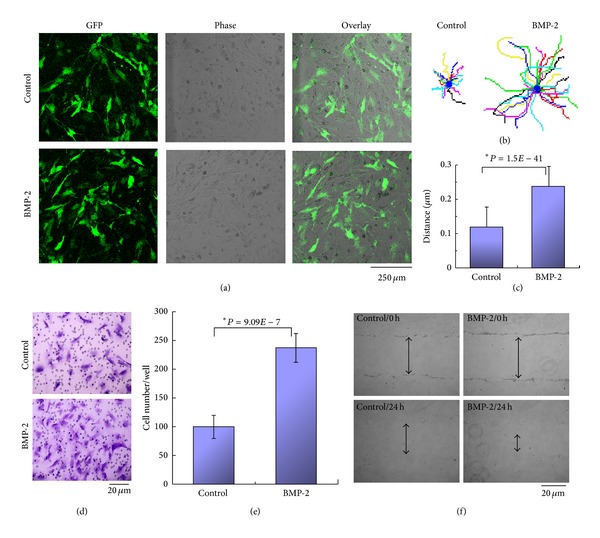
Adenoviral-mediated overexpression of BMP-2 and its effect upon cellular motility in both uni- and multicell populations. (a) GFP expression of adenoviral transfected rat VSMCs assessed by confocal microscopy. Infection efficiency exceeds 90%. (b) Rat VSMCs migration traced by time-lapse video microscopy. Sixteen representative paths for each treatment originated from a common point, in stellate fashion. Faster migration produced larger stars. Cellular motility was recorded on indicated substrates for 6 hours. (c) Movement assay: mean velocity determined by time-lapse recording via ImageJ software. Overexpression of BMP-2 approximately doubled migratory capacity. (d) Boyden chamber assay revealed increased cellular migration in adenoviral-transfected cells overexpressing BMP-2 compared to vector-only cells. (e) Quantification of migration of BMP-2 overexpressing transfected cells; values represent the mean ± SD of independent experiments. (f) Wounding assay, assessing motility, and spread potential of a cellular population. VSMCs cells were virally-transfected to overexpress BMP-2. Both at the time of cellular culture incision and 48 hours after, the state of wound closure was assessed by phase-contrast microscopy. Arrows indicate distance from incision edges. *Student's *t*-test: *P* < 0.01 versus control.

The adenoviral vector pAV.EX1d-CMV with cloned construct myc/IRES/EGFP was employed to overexpress BMP-2 in VSMCs. After purification, adenoviral titer was amplified in the recombinant adenoviral pAV-EX1d system, reaching 10^8^ transducing units/mL (physical viral particle concentration vp/mL). AGFP protein detectable in coprimary culture of infected rat VSMCs confirmed an expression ratio exceeding 90% (Figure 1(a)). Single rat VSMC migration traced by time-lapse video microscopy revealed BMP-2 overexpressing VSMCs are faster than those infected by vector alone. Swifter VSMC migration resulted in larger spanning stellate “star” formations (Figure 1(b)). The average distance travelled by a moving single cell in a six-hour observation period was confirmed every 15 minutes (BMP-2 travelled 0.24 ± 0.2 *μ*m versus control: 0.12 ± 0.1 *μ*m, Figure 1(c)). Migratory cell mounts of rat VSMCs overexpressing BMP-2 were compared to control cells in a Boyden chamber assay (Figure 1(d)). BMP-2 overexpression increased cellular intensity 130%, compared to vector alone (*P* < 0.01, Figure 1(e)). The wounding assay demonstrated BMP-2 influenced cellular population movement as well within 48 hours (BMP-2: 93.3 ± 17.8 *μ*m versus control: 43.7 ± 16.7 *μ*m, *P* < 0.01, Figure 1(f)).

### 3.2. BMP-2 Increases Motility of Both Unicellular and Multicellular VSMC Populations in Dose-Dependent Manner

**Figure 2:**
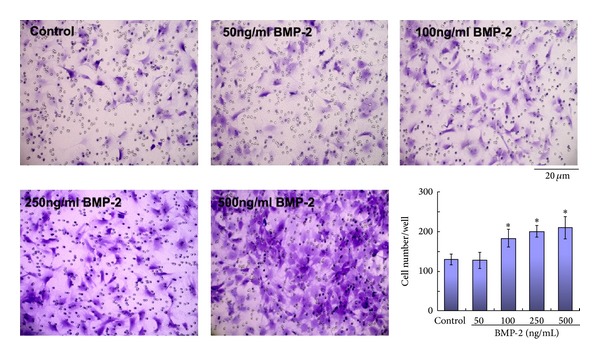
Dose-dependent effect of BMP-2 upon cellular motility. A Boyden chamber assay was performed upon rat VSMCs treated by exogenous BMP-2 concentrations. *Student's test: *P* < 0.01 versus control.

VSMCs were treated with varying BMP-2 concentrations (ranging from 50 to 500 ng/mL) via Boyden chamber for 48 hours.  Figure 2 demonstrates VSMCs exhibit a dose-dependent migratory effect in response to BMP-2 concentrations exceeding 100 ng/mL. Time-lapse video microscopy and wounding assay, respectively, demonstrated unicellular and multicellular VSMC populations responded in dose-dependent manner to BMP-2 (Table 2).

### 3.3. Identification of Genes Regulated by BMP-2 Overexpression

**Figure 3:**
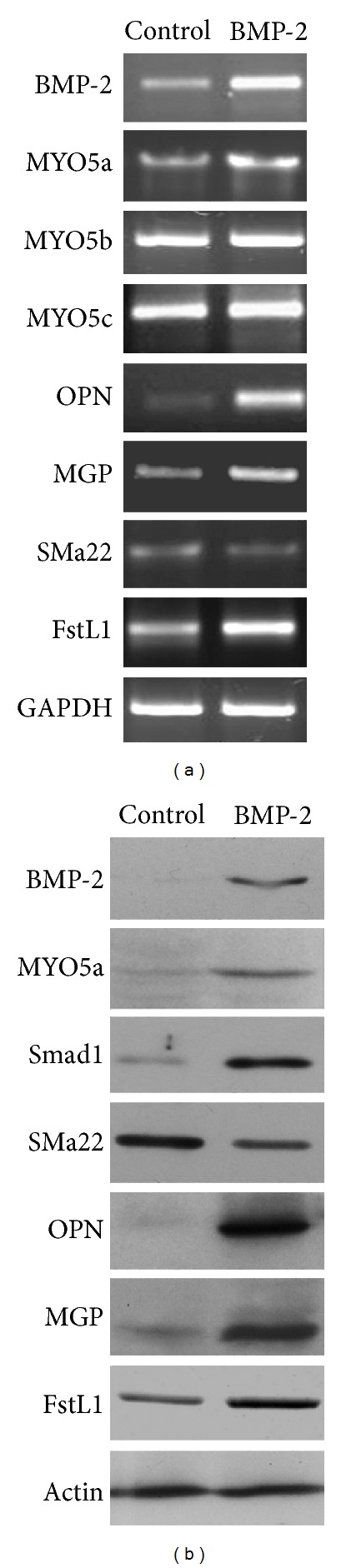
Gene chip array analysis demonstrating various gene and protein levels after gene array analysis. (a) Gene expression of myosin Va, myosin Vb, myosin Vc, OPN, MGP, SMa22, FSTL1, and GAPDH via RT-PCR. (b) Protein levels of BMP-2, myosin Va, Smad1, OPN, MGP, FSTL1, and pan-actin via Western analysis.

To gain mechanistic insight concerning BMP-2-mediated VSMC migration facilitation, microarray analyses were performed to determine global gene expression changes in BMP-2 overexpressing VSMCs. In VSMCs overexpressing BMP-2, 554 genes were downregulated, and 437 genes were upregulated. Not surprisingly, genes involved in the BMP signaling pathway (such as Fstl1, Fstl3, Smad1, and Msx1) were among those upregulated during BMP-2 overexpression. Although our gene chip array determined myosin Va was consistently upregulated during BMP-2 overexpression, myosin Vb and Vc were not detected. In addition, expression of smooth muscle alpha-actin, a characterizing marker of the systole phenotype, remained unchanged. The mRNA and protein levels of OPN and MGP, characterizing markers of the diastolic phenotype, were markedly altered (Figures 3(a) and 3(b)).

### 3.4. BMP-2 Overexpression Increases Myosin Va Expression

**Figure 4:**
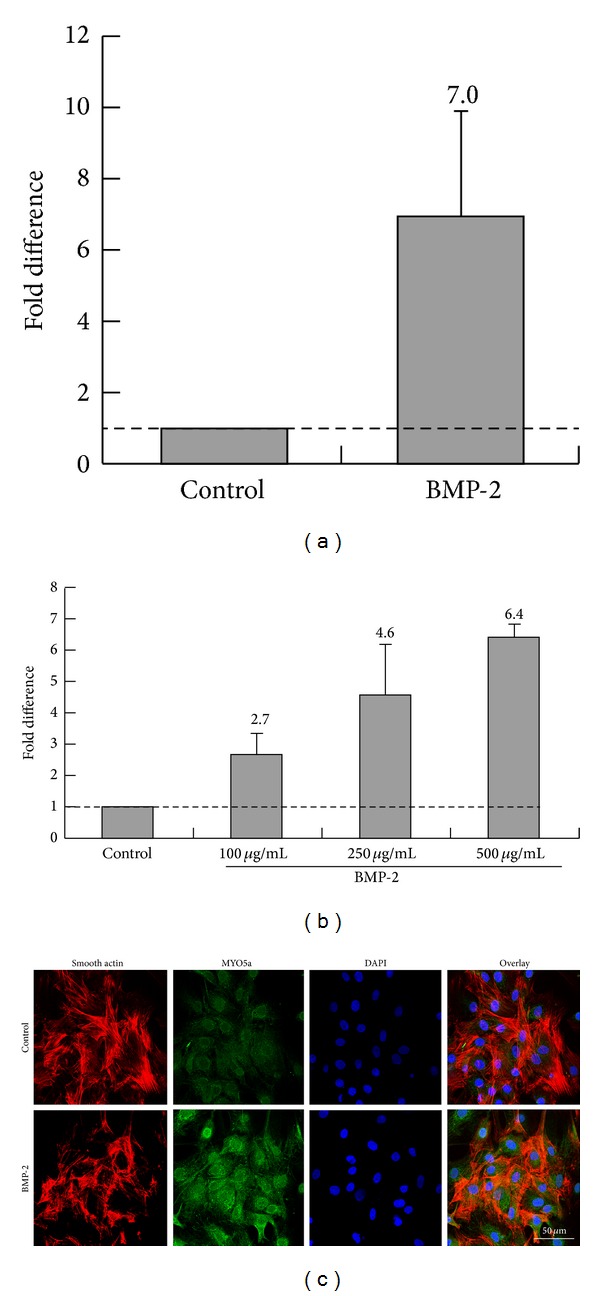
Myosin Va expression regulated by BMP-2. (a) qRT-PCR analysis revealed mRNA upregulation of the myosin Va gene associated with adenoviral-mediated BMP-2 overexpression in rat VSMCs. (b) Myosin Va expression reflects a dose-dependent response to BMP-2 administration. (c) Immunofluorescent assay characterizes actin and myosin Va expression. Myosin Va proteins were abundantly distributed in the cytoplasm of cells binding closed actin in BMP-2 overexpressing VSMCs, compared to control (which manifested only weakly positive signals).

Via RT-PCR, we determined adenoviral-mediated BMP-2 overexpression significantly increased myosin Va mRNA sevenfold compared to control (Figure 4(a)), in a dose-dependent manner (Figure 4(b)). We then confirmed BMP-2 stimulation increased myosin Va protein expression by characterizing actin and myosin Va expressing VSMCs by immunofluorescence analysis. Representative photographs in Figure 4(c) display abundantly distributed myosin Va protein binding actin in the cytoplasm of VSMCs overexpressing BMP-2, compared to the weakly positive signals detected in control cells. Together, these results strongly support augmented myosin Va expression during BMP-2 overexpression.

### 3.5. siRNA-Mediated knockdown of VSMC Myosin Va Inhibits Migration

**Figure 5:**
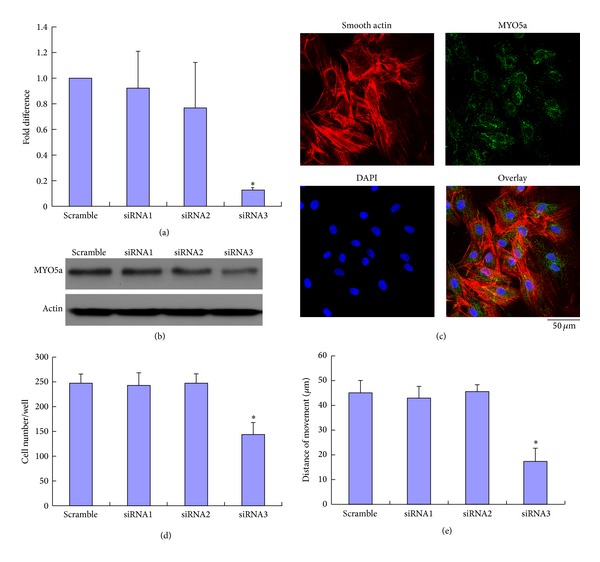
Effects of siRNA-mediated knockdown of myosin Vain rat VSMCs subjected to BMP-2 (250 ng/mL). (a) qRT-PCR analyses, demonstrating varying siRNA-mediated suppression of myosin Va mRNA expression. siRNA3 construct inhibited myosin Va mRNA expression to 0.13-fold of scramble levels. (b) Western blot demonstrating siRNA-3 construct inhibits the most myosin Va of all three generated siRNA constructs. (c) Myosin Va expression, determined by immunofluorescence, inhibited by siRNA-3. (d) Unicellular motility detected by Boyden chamber assay. (e) Multicellular population motility determined by wounding assay. Both (d) and (e) demonstrate that siRNA3 significantly inhibits migration compared to scramble control (unicellular motility 144 ± 25 versus 248 ± 20 cells/well, multicellular population 17.5 ± 5 *μ*m versus 45.2 ± 4.8 *μ*m). *Student's *t*-test: *P* < 0.01 versus control.

We generated three myosin Va-specific siRNA constructs. Compared to control or other generated siRNA constructs, siRNA-construct-3 (henceforth termed siRNA-3) significantly reduced the expression of both myosin Va mRNA (Figure 5(a)) and protein (Figure 5(b)). siRNA-mediated knockdown of myosin Va decreased its expression in locations known to be detectable by immunofluorescence assay in VSMCs simulated by BMP-2 (Figure 5(c)). We next determined the functional consequence of myosin Va knockdown in the setting of BMP-2 stimulation. VSMCs subjected to myosin Va-knockdown by siRNA-3migrate significantly slower compared to control or VSMCs subjected to siRNA constructs 1 or 2 (Figures 5(d) and 5(e)). These results suggest myosin Va may have significant role in BMP-2-mediated acceleration of VSMC migration.

### 3.6. Erk1/2 Modulates Myosin Va Expression by BMP-2

**Figure 6:**
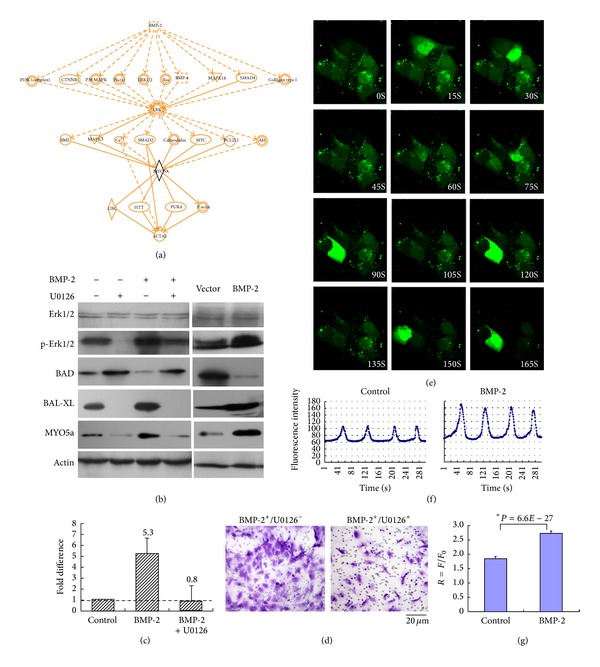
BMP-2-mediated regulation of myosin Va may be modulated by Erk signaling with Ca^2+^ involvement. (a) IPA software analysis of the relationships and interactions between BMP-2, Erk1/2, myosin Va, and actin. Although communication between the genes of these proteins genes has previously been reported, heretofore, a direct interaction between BMP-2 and myosin Va was not clarified. (b) Western demonstrates the presence of key proteins involved with myosin Va and Erk signaling in the lysates of rat VSMCs either adenovirally transfected to overexpress BMP-2 or treated with exogenous BMP-2 (250 ng/mL) for 48 hours. (c) qRT-PCR analysis demonstrating myosin Va gene mRNA was upregulated in VSMCs treated with 250 ng/mL BMP2 for 48 hours and downregulated in the presence of Erk1/2 inhibitor U0126 (dose 5 *μ*M). (d) Migration of VSMCs subjected to identical conditions was detected by Boyden chamber assay (BMP-2^+^ alone: 253 ± 29 versus BMP-2^+^/U0126^+^: 176 ± 11). (e) Oscillatory increase of intracellular Ca^2+^ concentration ([Ca^2+^]^i^) within individual cells of the colony. Chronological image sequence goes from left to right at 1.5-second intervals over 5 minutes. (f) Oscillations exhibited a mean interval of intracellular Ca^2+^ concentration (BMP-2 concentration 250 ng/mL, *n* = 18). (g) Fluorescence (*F*/*F*_0_) of individual cells, values represent mean ± SD; *Student's *t*-test: *P* < 0.01).

The signaling mechanisms responsible for the effects of BMP-2 overexpression upon VSMC migration are unclear. Software (IPA, Ingenuity Sys) analysis revealed the relationship and interaction between BMP-2, Erk1/2, myosin Va, and actin (Figure 6(a)). We investigated the degree of Erk1/2 stimulation in response to BMP-2 doses referenced in previous studies [23]. VSMCs were either treated with exogenous 250 ng/mL BMP-2 for 48 hours or adenovirally transfected to overexpress BMP-2. VSMCs were consequently exposed to Erk1/2 inhibitor U0126. Erk1/2 activation was determined by Western blot. Erk1/2 inhibitor significantly increased BAD protein production (typically downregulated by BMP-2) and significantly decreased Bcl-xl and myosin Va expression (typically upregulated by BMP-2) (Figure 6(b)). qRT-PCR demonstrated upregulation of myosin Va gene mRNA in rat VSMCs cells treated with 250 ng/mL BMP-2 for 48 hours, an effect blocked by 5 *μ*M U0126 (Figure 6(c)). U0126 attenuated BMP-2 augmented VSMC motility (Figure 6(d)). Together, these results suggest Erk1/2 activity is essential for modulating myosin Va expression by BMP-2.

### 3.7. BMP-2 Increases Intracellular [Ca^2+^]^i^Oscillation

Calcium (Ca^2+^) plays a pivotal role in physiological biochemistry, acting as a second messenger in signal transduction pathways, involved with neurotransmitter release, all muscular cell type contraction, and fertilization [24, 25]. We investigate the potential involvement of Ca^2+^ in the signaling mechanism between BMP-2 and Erk1/2 by recording intracellular [Ca^2+^]^i^ oscillations within VSMCs. After loading the Ca^+^-sensitive dye Fluo-4 AM, we monitored the time-dependent change of [Ca^2+^]^i^ within a colony's individual cells. Figure 6(e) demonstrates a representative VSMC colony, in which individual cells exhibit spontaneous Ca^2+^ oscillation. Without external Ca^2+^ supplementation, prolonged BMP-2 administration activated a large transient increase in Ca^2+^, followed by a burst of Ca^2+^ spikes. The average amplitude of spontaneous oscillations increased intracellular Ca^2+^ concentration ([Ca^2+^]^i^) (Figure 6(f)). Individual cell fluorescence (*F*/*F*_0_) plotted as a function of time demonstrates oscillatory changes in [Ca^2+^]^i^. Such [Ca^2+^]^i^ oscillations significantly increased in cells subjected to 250 ng/mL BMP-2 (Figure 6(g)). These data suggest BMP-2-induced Ca^2+^ oscillations are generated largely by a “cytosolic oscillator” in VSMCs. BMP-2-mediated regulation of myosin Va may be modulated by Erk signaling with Ca^2+^ involvement.

## 4. Discussion

We have made several important observations in the current study. Firstly, we demonstrated BMP-2 overexpression in VSMCs augments both unicellular and multicellular population motility. Secondly, we demonstrated BMP-2 overexpression increases the expression of myosin Va but not myosin Vb or Vc. Finally, we provide mechanistic evidence of ERK1/2 involvement in the modulation of myosin Va expression by BMP-2, with Ca^2+^ involvement.

Myosin Va directly assembles actin into antiparallel bundles [26, 27] localized at the leading edge of membrane ruffles [28]. A veritable actin-based motor protein, myosin Va, functions as an intracellular vesicle and organelle transporter and delivers cargo critical for maintaining cellular movement, thereby supporting cellular migration. Heretofore, whether myosin Va regulates VSMC migration was unknown. In this study, we demonstrate the abundant distribution of myosin Va binding actin in the cytoplasm of BMP-2 overexpressing VSMCs, compared to control cells. We also demonstrate knockdown of myosin Va inhibits VSMC motility, suggesting myosin Va is requisite for VSMC migration. More detailed analysis is required to elucidate how myosin Va specifically affects cellular motility, but it is plausible myosin Va participates in cell cytoskeleton reorganization, an essential event driving cellular movement [13, 29].

We clearly demonstrate BMP-2 overexpression stimulates cellular migration. We employed IPA software to analyze the relationships and interactions among BMP-2, Erk1/2, Myosin Va, and Ca^2+^, but previous reports have not revealed any direct relationship between BMP-2 and myosin Va [30–32]. Erk1/2 inhibition not only blocked BMP-2-mediated downregulation of Bcl-xl protein production but also significantly decreased expression of both myosin Va and BAD. Myosin Va may mediate the functions of Bcl-xl by promoting islet cell migration and invasion [15]. We have demonstrated BMP-2 modulates myosin Va expression in VSMCs via Erk1/2 but cannot preclude the involvement of other contributory factors regulating the expression of myosin Va, such as gene regulatory elements like promoters and miRNAs. Further work is necessary to fully characterize the myosin Va promoter.

Spontaneous calcium oscillations occur in cells originating from excitable tissues of muscular, neuronal, and embryonic stem origin [33–35]. Calcium is an important second messenger regulating both intracellular and extracellular communications. The amplitude and duration of calcium transients can promote the expression of specific genes. Myosin Va is necessary for localization of IP3 receptors, which may connect with intracellular Ca^2+^ [24, 25]. In the current study, we observe the presence of spontaneous [Ca^2+^]^i^ oscillations in cultured VSMCs, further activated in the presence of BMP-2. The Erk signaling pathway can be stimulated by increased [Ca^2+^]^i^ oscillations, augmenting Erk1/2 phosphorylation [36, 37], supported by our results (Figure 6). Additional studies specifically defining the precise roles of Ca^2+^ as a second messenger in this system are ongoing.

VSMCs can change phenotype* in vivo* depending upon functional demands and can be contractile, proliferative, migratory, and/or synthetic [38, 39]. We demonstrate that BMP-2 overexpression increased expression of synthetic phenotypic markers OPN and MGP and decreased contractile phenotypic marker SM22*α*. The present study advances the notion BMP-2 may modulate SMC phenotype towards a synthetic state. Further studies are necessary to determine whether Ca^2+^ oscillations may have phenotypic implications.

In summary, our study demonstrates BMP-2 enhances VSMC migration via Erk1/2 signaling activation, which regulates myosin Va expression. Inhibition of BMP-2-induced myosin Va expression may represent a potential future therapeutic strategy attenuating atherosclerosis.

## Figures and Tables

**Table:** (a)

Gene name	Sense	Antisense	Size (bp)
*BMP-2 *	5′-TATATGCTCGACCTGTACCG-3′	5′-CTTCCTGCATTTGTTCCCGA-3′	247
*Myosin Va *	5′-CACCTACGGAACCCTGACAT-3′	5′-TGCAAAGATGTGAGGGTCCA-3′	243
*Myosin Vb *	5′-GGCTGAACTAACCAAGGACT-3′	5′-GGCAACAAGCACAATTCCAC-3′	256
*Myosin Vc *	5′-GAAGATGGAAGGAAAGCAGG-3′	5′-TCCTCAAGATCCCTGTTTTC-3′	255
*SPP1 *	5′-TGGCTTACGGACTGAGGTCA-3′	5′-GACCTCAGAAGATGAACTC-3′	486
*MGP *	5′-ATCCTGGCTGCGCTGGCCGTG-3′	5′-GAAGTAGCGGTTGTAGGCGGC-3′	264
*Sma22 *	5′-AAGCCAGTGAAGGTGCCTGAG-3′	5′-TTGAAGGCCAATCACGTGCTT-3′	311
*Fstl1 *	5′-GTGGCAGTAATGGCAAGAC-3′	5′-GTACTTGTCTAGGATCTCAC-3′	260
*GAPDH *	5′-AAGAAGGTGGTGAAGCAGG-3′	5′-ACCCTGTTGCTGTAGCCATA-3′	197

**Table:** (b)

Name	Vender	Cat. no.	Species	Dilution
BMP-2	Bioworld	BS3473	Rabbit monoclonal IgG	1 : 500
MYO5a	Sigma	Sab2501441	Goat polyclonal IgG	1 : 1000
Smad1	Cell Signaling	9516	Rabbit monoclonal IgG	1 : 1000
Sm22	Epitomics	S2112	Rabbit polyclonal IgG	1 : 1000
OPN	Bioworld	BS1264	Rabbit monoclonal IgG	1 : 1000
MGP	Abgent	Ap11953	Rabbit polyclonal IgG	1 : 200
FSTL1	Abcam	Ab71548	Rabbit polyclonal IgG	1 : 1000
Actin	Anbo	C0124	Rabbit polyclonal IgG	1 : 5000

**Table 2:** BMP-2 increased uni- and multicellular population motility.

	Control	50 ng/mL	100 ng/mL	250 ng/mL	500 ng/mL
Signal cell movement distance (um/15 min)	0.112 + 0.01	0.134 + 0.01	0.165 + 0.02	0.202 + 0.05	0.221 + 0.028
*P* value with ctrl		0.08	0.0021*	0.0003*	0.00032*
Populations movement distance (um/24 h)	40.5 + 10.2	48.0 + 12.6	56.9 + 10.3	76.7 + 9.6	80.1 + 13.2
*P* value with ctrl		0.32	0.034*	0.0004*	0.0007*

Compared to control group, Student's test: *P < 0.01.
